# Special Pathology and Diagnostics

**Published:** 1882-01

**Authors:** 


### BOOK NOTICES, REVIEWS, ETC.

Special Pathology and Diagnostics, with Therapeutic Hints By *C. G. Raue, M. D.* Second edition, rewritten and enlarged, pp. 1072, large 8vo.; price $8. Boericke and Tafel: New York and Philadelphia. London: Homœopathic Publishing Co., No. 2 Finsbury Circus. 1882.

This edition of Dr. Raue's book is in every respect an improvement upon the former edition. If the first was good, the second is better, and will be found of great practical value. As to the scope and plan of this edition, we need only say, they are the same as in the former, only enlarged and improved. The "Therapeutic Hints" are more numerous, clearer and fuller. The pathology is brought up to date, with all *modern improvements!*

In his preface, the author says: "The first edition has become old; it needed renovation. The pathological views had changed so grievously since its appearance that a re-statement of the same throughout the work became a necessity. Not so, however, the therapeutic hints. They are as true to-day as they were when written years ago and, I am happy to say, have been reliable guides at the bedside to many physicians; also a fruitful source, acknowledged or not, to many writers in journals and of books. What I had to do with these hints is this: To express their meaning still more accurately, to enlarge their spheres, and to add such new facts as the experience of others and my own would admit. This has augmented to a considerable extent even the therapeutic part of the work, and thus I may state in truth, that this second edition is re-written for the most part, that it is greatly enlarged, and, I hope, greatly improved."

Pathological views are based on speculative theory; hence they changed so grievously that a re-statement of them became necessary. Homœopathic therapeutics is based on *law;* hence we read: "Not so, however, the therapeutic hints. They are as true to-day as when written years ago." Let the advocates of a pathological basis for homœopathic therapeutics ponder well these words of Dr. Raue.

